# Structurally triggered metal-insulator transition in rare-earth nickelates

**DOI:** 10.1038/s41467-017-01811-x

**Published:** 2017-11-22

**Authors:** Alain Mercy, Jordan Bieder, Jorge Íñiguez, Philippe Ghosez

**Affiliations:** 1 Theoretical Materials Physics, Q-MAT, CESAM, University of Liège, B-4000 Liège, Belgium; 2 CEA DAM-DIF, F-91297 Arpajon, France; 3 Materials Research and Technology Department, Luxembourg Institute of Science and Technology (LIST), 5 avenue des Hauts-Fourneaux, L-4362 Esch/Alzette, Luxembourg

## Abstract

Rare-earth nickelates form an intriguing series of correlated perovskite oxides. Apart from LaNiO_3_, they exhibit on cooling a sharp metal-insulator electronic phase transition, a concurrent structural phase transition, and a magnetic phase transition toward an unusual antiferromagnetic spin order. Appealing for various applications, full exploitation of these compounds is still hampered by the lack of global understanding of the interplay between their electronic, structural, and magnetic properties. Here we show from first-principles calculations that the metal-insulator transition of nickelates arises from the softening of an oxygen-breathing distortion, structurally triggered by oxygen-octahedra rotation motions. The origin of such a rare triggered mechanism is traced back in their electronic and magnetic properties, providing a united picture. We further develop a Landau model accounting for the metal-insulator transition evolution in terms of the rare-earth cations and rationalizing how to tune this transition by acting on oxygen rotation motions.

## Abstract (web summary)

Applications of rare-earth nickelates are hampered by lack of global understanding of the interplay among various degrees of freedom. Here, Mercy et al. propose that the metal-insulator transition of nickelates arises from the softening of an oxygen breathing distortion, providing a united picture of electronic, structural and magnetic properties.

## Introduction

**Fig. 1:**
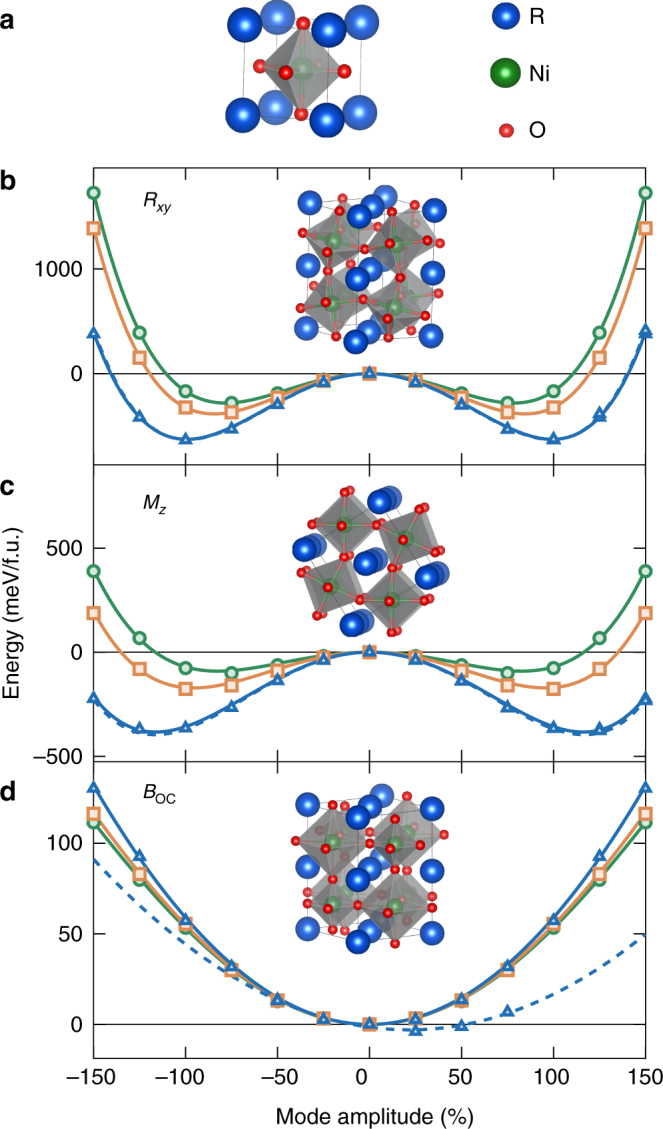
Reference cubic perovskite structure and most relevant atomic distortions. **a** Sketch of the reference $Pm\bar 3m$ cubic perovskite cell of *R*NiO_3_ compound with *R* at the corner, Ni at the center, and O atoms at the middle of the faces, forming corner-shared octahedra surrounding the B atoms. Starting from this reference, three main atomic distortions drive the system successively to the *Pbnm* and then *P*2_1_/*n* phases: **b** anti-phase rotations of oxygen octahedra of same amplitudes about *x*- and *y*-axis (*R*_*xy*_), **c** in-phase rotations of oxygen octahedra about *z*-axis (*M*_*z*_), **d** breathing of the oxygen octahedra (*B*_OC_). The energy wells associated to the freezing of individual distortion of increasing amplitude in the cubic cell are shown for different *R* cations, associated to distinct tolerance factor *t*, and either a ferromagnetic (full line) or E′-type antiferromagnetic (dashed line, only for *R* = *Y*) spin arrangement: YNiO_3_ (*t* = 0.920, blue); GdNiO_3_ (*t* = 0.938, orange) and SmNiO_3_ (*t* = 0.947, green). The atomic distortions are normalized to their amplitude in the *P*2_1_/*n* AFM-E′ ground state of YNiO_3_. Calculations are done for each compound in a cubic cell that has the same volume as the ground state

First synthesized in 1971^1^, rare-earth nickelates (*R*NiO_3_, with *R* = rare earth) are appealing for various applications^2, 3^, and the possibility to tune their properties in epitaxial films and heterostructures^4^ has recently fueled an even larger interest^5–7^. *R*NiO_3_ compounds belong to the family of perovskite oxides with a reference $Pm\bar 3m$ cubic structure (Fig. 1a), nevertheless not directly observed. Apart for LaNiO_3_, which always keeps a metallic $R\bar 3c$ phase and will not be further discussed here, all *R*NiO_3_ adopt at reasonably high temperature a metallic *Pbnm* orthorhombic phase^8^. This phase, rather ubiquitous^9^ amongst perovskites with small Goldschmidt tolerance factor^8^, *t*, corresponds to a distortion of the cubic structure arising from the appearance of combined anti-phase rotations of the oxygen octahedra along the *x* and *y* directions, *R*_*xy*_ (Fig. 1b), with the same amplitude *Q*_R_ and in-phase rotations of the oxygen octahedra along *z*, *M*_*z*_ (Fig. 1c), with amplitude *Q*_M_.

In this phase, all Ni atoms are equivalent and formally in a Jahn-Teller active *d* ^7^ (likely $t_{{\mathrm{2g}}}^6e_{\mathrm{g}}^1$ low spin) configuration. Surprisingly, although compatible with the *Pbnm* symmetry, cooperative Jahn-Teller distortions do not appear. Instead, at a temperature *T*_MI_, which strongly evolves with the *R* cation (i.e., with *t*)^10^, *R*NiO_3_ compounds exhibit an electronic metal-insulator transition (MIT), which was shown to be concurrent with a structural transition from *Pbnm* to monoclinic *P*2_1_/*n* symmetry^11^. This symmetry lowering is accompanied with the appearance of a breathing distortion of the oxygen octahedra, *B*_OC_ (Fig. 1d), which alternatively expand and contract with amplitude *Q*_B_, according to a rock-salt pattern. This gives rise to two types of Ni sites with long and short Ni–O bonds, respectively.

At low temperature (100–200 K), *R*NiO_3_ compounds finally show a magnetic phase transition toward an unusual E′-type antiferromagnetic (AFM) spin order identified by a Bragg vector **q** = (1/4, 1/4, 1/4) in pseudocubic notation^11–13^. For large cations (*R* = Nd and Pr), the Néel temperature *T*_N_ = *T*_MI_ and the system goes directly from paramagnetic metal (PM-M) to AFM insulator (AFM-I). For smaller *R* cations, *T*_N_ is much lower than *T*_MI_; the two phase transitions are decoupled and the system goes through an intermediate paramagnetic insulating phase (PM-I).

The origin of the MIT has been widely debated in the literature^14–19^. It was sometimes interpreted as a charge disproportionation at Ni sites^20^: 2(*d* ^7^) → *d* ^8^ + *d* ^6^. However, the importance of Ni 3*d*-O 2*p* hybridization—i.e., transfer of electrons from O to Ni and formation of oxygen holes $\left( {\underline L } \right)$ keeping Ni in a *d* ^8^ configuration (i.e., $d^{8 - n} \approx d^8\underline L ^n$)—was evoked early on^15^. Nowadays, the MIT is usually viewed as a charge ordering of the type $2\left( {d^8\underline L ^1} \right) \to \left( {d^8} \right) + \left( {d^8\underline L ^2} \right)$ ^17, 21, 22^. In this scenario, *B*_OC_ appears important to stabilize the charge ordering and open the gap. As suggested in ref. ^23^ and confirmed from statistical correlation techniques^24^, *R*_*xy*_ and *M*_*z*_ are also expected to play an active role. However, a complete picture linking electronic, structural and magnetic properties is yet to emerge.

Unlike recent theoretical studies, which were focusing specifically on the electronic properties^17–19, 21^, we investigate here self-consistently the electronic and structural degrees of freedom of *R*NiO_3_ compounds from density functional theory calculations (DFT, see Methods). Specific care was given to the validation of our approach, which turns out to provide an unprecedented agreement with experimental data. Focusing on YNiO_3_, we show (Supplementary Notes 1–4) that not only the atomic structure but also the AFM-E′ ground state, the estimated *T*_N_ and the electronic bandgap of the insulating phase are very accurately reproduced, making therefore our approach a method of choice to shed light on the interlink between these different features.

## Results

### Energetics of individual lattice distortions

We start from the reference $Pm\bar 3m$ cubic structure. Inspection of the phonon dispersion curves (Supplementary Note 5) reveals dominant structural instabilities at R (*ω*_R_ = 310i cm^−1^) and M (*ω*_M_ = 278i cm^−1^) points of the Brillouin zone (BZ), which are associated, respectively, to the *R*_*xy*_ and *M*_*z*_ distortions responsible for the *Pbnm* phase. These imaginary frequencies *ω*_i_ are linked to a negative energy curvature *α*_i_ at the origin $\left( {\alpha _{\mathrm{i}} \propto \omega _{\mathrm{i}}^2 < 0} \right)$ and to a typical double-well (DW) shape of the energy when freezing *R*_*xy*_ and *M*_*z*_ distortions of increasing amplitude within the cubic structure, as illustrated in Fig. 1. These wells are nearly independent of the spin order but strongly evolve with the *R* cation: they become shallower when *R* size increases, consistently with a reduction of the related distortion amplitudes in the *Pbnm* phase.

In contrast, the *B*_OC_ motion, corresponding to another phonon at R, is stable and extremely stiff (in fact the stiffest mode with *ω*_B_ = 700 cm^−1^), in line with the single-well (SW) shape illustrated in Fig. 1. Decreasing *R* cation size tends to stabilize slightly further *B*_OC_, in apparent contradiction with the observed increase of *T*_MI_. As illustrated for YNiO_3_, switching from ferromagnetic (FM) to AFM-E′ spin order reduces slightly the curvature but does not reverse it; instead it shifts the SW to lower energy^13^, yielding a finite *Q*_B_ at the minimum. Although *B*_OC_ tends to make the system insulating, this amplitude (25% of ground state’s value) is however not large enough to open a gap (more than 75% would be required). This shows that *B*_OC_ and the magnetic order alone cannot explain the MIT by themselves.

### Mode coupling and triggered mechanism

**Fig. 2:**
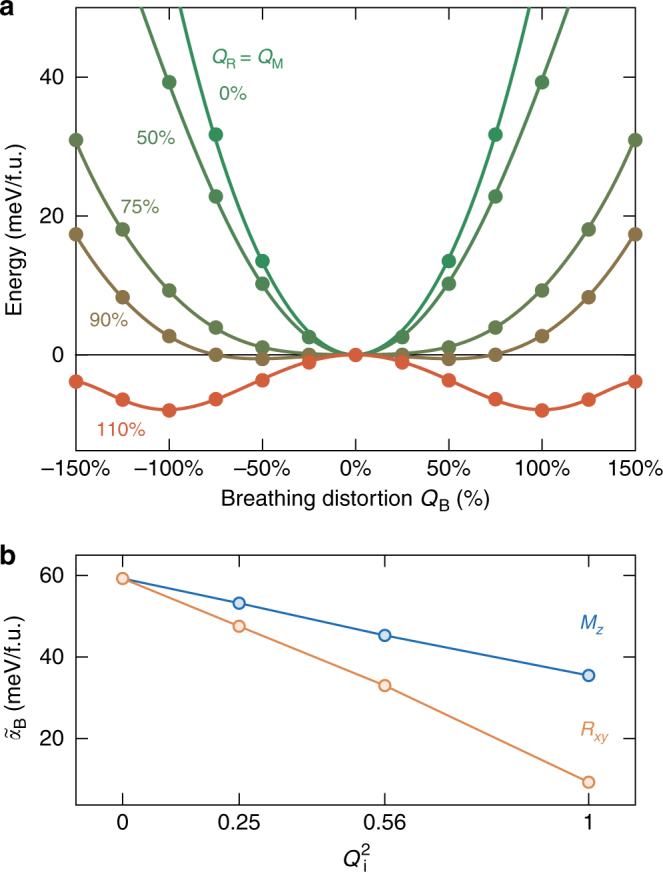
Triggered mechanism. **a** Evolution of the energy *E* in terms of the amplitude of the breathing distortion (*Q*_B_) for fixed amplitudes of oxygen rotations (*Q*_R_ = *Q*_M_ from 0 to 110%) in the FM cubic cell of YNiO_3_ (same volume as the ground state). It highlights the softening of the energy well associated to *B*_OC_, triggered by the oxygen rotations *R*_*xy*_ and *M*_*z*_. **b** Linear evolution of the energy curvature at the origin, $\tilde \alpha _B$ along *Q*_B_, in terms of the square of the amplitude of the individual distortions *Q*_R_ (orange) and *Q*_M_ (blue)

Our central result is presented in Fig. 2 where we report in panel a the evolution of the *B*_OC_ energy well of YNiO_3_ at various fixed amplitudes of oxygen rotation motions. It highlights that although initially stable (SW), *B*_OC_ is progressively destabilized (DW) by the appearance of *R*_*xy*_ and *M*_*z*_. As illustrated in panel b, *α*_B_ is renormalized into $\tilde \alpha _{\mathrm{B}}$, which evolves linearly with $Q_{\mathrm{R}}^2$ and $Q_{\mathrm{M}}^2$. The slope associated to *Q*_R_ is twice as large as that related to *Q*_M_, attesting that each of the three individual rotations similarly affects *B*_OC_. This behavior arises from the presence in the energy expansion of cooperative (*λ* < 0) bi-quadratic coupling terms between *B*_OC_ and oxygen rotations ($E \approx \lambda _{{\mathrm{Bi}}}Q_{\mathrm{B}}^{\mathrm{2}}Q_{\mathrm{i}}^{\mathrm{2}}$, i = M,R), which, being the lowest-order couplings allowed by symmetry, should give rise to the appearance of *B*_OC_ through a triggered phase transition according to Holakovsky^25^. The same behavior is observed independently of the magnetic order (Supplementary Note 8). From now we focus on representative FM results while coming back to the role of magnetism later.

### Landau model and phase diagram

To further assess the relevance of such a triggered mechanism in nickelates, we built a Landau model, including *R*_*xy*_, *M*_*z*_, and *B*_OC_ degrees of freedom^24^, restricting ourselves to lowest coupling terms and assuming temperature dependence of the oxygen rotations only:

1 $$\begin{array}{*{20}{l}} E \hfill & \hskip-8pt = \hfill &\hskip-7pt {\gamma _{\mathrm{R}}\left( {T - T_{{\mathrm{0R}}}} \right)Q_{\mathrm{R}}^{\mathrm{2}} + \beta _{\mathrm{R}}Q_{\mathrm{R}}^4 + \lambda _{{\mathrm{BR}}}Q_{\mathrm{B}}^2Q_{\mathrm{R}}^2} \hfill \\ {} \hfill & + \hfill & {\gamma _{\mathrm{M}}\left( {T - T_{{\mathrm{0M}}}} \right)Q_{\mathrm{M}}^2 + \beta _{\mathrm{M}}Q_{\mathrm{M}}^4 + \lambda _{{\mathrm{BM}}}Q_{\mathrm{B}}^2Q_{\mathrm{M}}^2} \hfill \\ {} \hfill & + \hfill & { \alpha _{\mathrm{B}}Q_{\mathrm{B}}^2 + \beta _{\mathrm{B}}Q_{\mathrm{B}}^4 + \lambda _{{\mathrm{MR}}}Q_{\mathrm{M}}^2Q_{\mathrm{R}}^2.} \hfill \end{array}$$

Within this model, *R*_*xy*_ and *M*_*z*_ appear at *T*_0R_ and *T*_0M_, respectively. On cooling, they progressively develop within the *Pbnm* phase and renormalize the energy curvature *α*_B_ of *B*_OC_ as made clear when grouping the $Q_{\mathrm{B}}^2$ terms in Eq. (1):

2 $$\tilde \alpha _{\mathrm{B}} = \alpha _{\mathrm{B}} + \lambda _{{\mathrm{BM}}}Q_{\mathrm{M}}^2 + \lambda _{{\mathrm{BR}}}Q_{\mathrm{R}}^2$$

When reaching a critical amplitude at which $\tilde \alpha _{\mathrm{B}} = 0$, they trigger the appearance of *B*_OC_ and produce concurrent structural and metal-insulator phase transitions. The phase transition appears to be second order within this model, which is however too simple to be conclusive on this point (Supplementary Note 10).

**Fig. 3:**
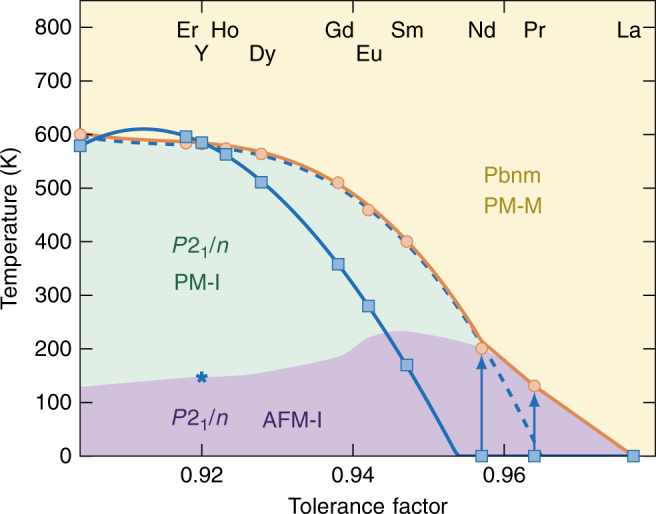
Nickelate phase diagram. Phase diagram of *R*NiO_3_ compounds in terms of their tolerance factor *t* and the temperature *T*. It includes three phases: a metallic *Pbnm* paramagnetic phase (PM-M, yellow area); an insulating *P*2_1_/*n* paramagnetic phase (PM-I, green area); and an insulating *P*2_1_/*n* E′-type AFM phase (AFM-I, magenta area). The yellow line and dots show the experimental evolution of *T*_MI_ with the tolerance factor *t*. The blue line and squares is the prediction of the simple Landau model fitted on the first-principles data (FM order). The dashed blue line is the fit of the Landau expression of *T*_MI_ (*t*) on experimental data. The blue star is the magnetic phase transition predicted for YNiO_3_ from first principles. The blue arrows indicate the correction to be applied on *T*_MI_ for large cations when properly incorporating the change of magnetic order

All parameters and their evolution with *R* were directly fitted from first principles; only Curie temperatures were uniformly scaled to reproduce the experimental *T*_MI_ of YNiO_3_ (Supplementary Note 8). The phase diagram of nickelates as predicted within this model is reported in Fig. 3. This figure demonstrates that the cooperative coupling of *B*_OC_ with *R*_*xy*_ and *M*_*z*_ is a key mechanism that, by itself, accounts for the experimentally observed evolution of *T*_MI_ with the tolerance factor.

Hence, the MIT in nickelates turns out to be a concrete example of triggered phase transition^26, 27^, a kind of transition never identified before in simple perovskites. Indeed, although bi-quadratic interactions are generic in this class of compounds, different distortions usually compete and exclude each other^9^. The cooperative coupling of *B*_OC_ with oxygen rotations pointed out here is therefore an unusual and intriguing feature, whose origin is now traced back in the electronic band structure.

### Electronic origin of the triggered mechanism

**Fig. 4:**
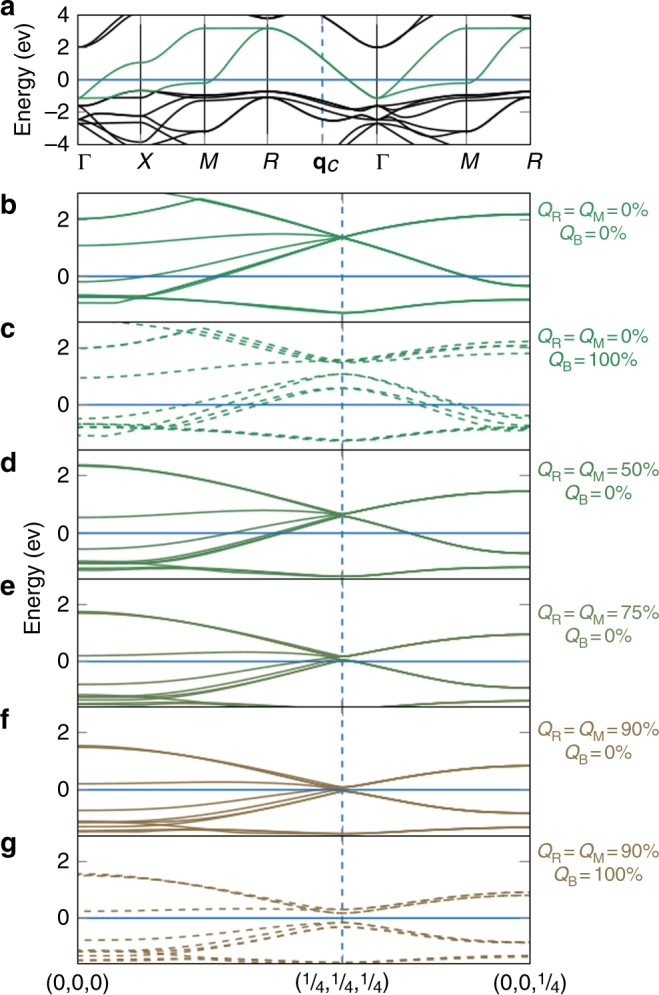
Electronic properties. **a** Electronic dispersion curves of YNiO_3_ along different high-symmetry line of the Brillouin zone of the $Pm\bar 3m$ phase (FM case, majority spins): Γ = (0, 0, 0), *X* = (1/2, 0, 0), *M* = (1/2, 1/2, 0), and *R* = (1/2, 1/2, 1/2). The Ni 3d *e*_g_ bands are highlighted in green. The Fermi energy corresponds to the horizontal blue line. The point **q**_c_ = (1/4, 1/4, 1/4) is located by vertical dashed blue lines. **b**–**g** Electronic dispersion curves around the Fermi energy *E*_f_ (FM order, majority spins) along high-symmetry points (coordinates in pseudocubic notations) in the Brillouin zone of the *Pbnm* or *P*2_1_/*n* 20-atom cell, in which bands have been folded respect to **a**. Consecutive panels show the evolution of the dispersion curves when freezing into the $Pm\bar 3m$ phase increasing amplitudes of oxygen rotations (*Q*_R_ = *Q*_M_ = **b** 0%, **d** 50%, **e** 75%, or **f** 90%, lines) and eventually adding the breathing distortion (*Q*_B_ = 100% and *Q*_R_ = *Q*_M_ = **c** 0% or **g** 90%, dashed lines)

In the cubic phase, as expected from the formal Ni 3*d* ^7^ $\left( {t_{{\mathrm{2g}}}^6e_{\mathrm{g}}^1} \right)$ occupancy, the Fermi energy *E*_f_ crosses levels of dominant Ni 3*d*-*e*_g_ character (i.e., anti-bonding Ni 3*d*-O 2*p* states); such levels form an isolated and rather dispersive set of two *e*_g_ bands, shifted above the *t*_2g_ levels by the crystal field (Fig. 4). Forcing into this cubic structure a *B*_OC_ distortion, associated to a phonon at **q**_R_ = (1/2, 1/2, 1/2), can open a gap at **q**_c_ = (1/4, 1/4, 1/4) within the *e*_g_ bands but well above *E*_f_ and without any direct impact on the metallic character and the occupied states. Nevertheless, the oxygen rotation motions substantially affect the *e*_g_ bands (Fig. 4), reducing their bandwidth and yielding a progressive down shift of the *e*_g_ levels at **q**_c_. As the rotations gradually increase and the bandwidth decreases, *B*_OC_ more substantially pushes down the electronic states around *E*_f_, providing a progressive gain of electronic energy, which, in turn, results in the softening of *ω*_B_. The critical rotation amplitude at which *B*_OC_ becomes unstable $\left( {\tilde \alpha_{\mathrm{B}} = 0} \right)$ is precisely that at which the *e*_g_ levels at **q**_c_ cross *E*_f_. At these amplitudes, the electronic system itself becomes unstable; the appearance of *B*_OC_ is favored and opens a gap at *E*_f_, making the system insulating. As such, the MIT can therefore be interpreted as a Peierls instability but one that is not initially present and has been triggered by oxygen rotations.

## Discussion

For compounds with small *R* cations, *Q*_R_ and *Q*_M_ are large and able to produce the MIT at relatively high temperatures, well above *T*_N_. For large cations (*R* = Nd, Pr), oxygen rotations are reduced and, from our Landau model (built on FM results), no more sufficient to trigger the MIT (Fig. 3). However, as previously mentioned, the AFM-E′ spin order is compatible by symmetry with *B*_OC_ and induces its appearance as an improper order (linear shift of *B*_OC_ SW, Fig. 1d). Hence, although not opening a gap in the cubic phase, the onset of the AFM-E′ order in the *Pbnm* phase of NdNiO_3_ and PrNiO_3_ promotes the occurrence of the MIT almost triggered by the rotations. In these compounds, we have therefore *T*_MI_ = *T*_N_; the transition is more abrupt and first-order^8^. Such active role of magnetism for large cations is supported by the experimental results and discussion in ref. ^28^. It is also confirmed by our first-principles calculations on NdNiO_3_, showing that the predicted *T*_MI_ is rescaled when including the change of magnetic order: while the system prefers to stay in the *Pbnm* metallic phase when imposing a FM order, it switches to the *P*2_1_/*n* phase when adopting a AFM-E′ order. The cooperative effect of the magnetic order remains true for small cations but without any impact on *T*_MI_ (>*T*_N_).

In conclusion, the concurrent electronic and structural transitions at *T*_MI_ in *R*NiO_3_ compounds take the form of a Peierls instability, which, primarily, is structurally triggered by the oxygen rotation motions *R*_*xy*_ and *M*_*z*_ and, eventually, is further assisted by the appearance of the E′-type AFM magnetic ordering. Our Landau model, and its possible extension to incorporate explicitly strain degrees of freedom neglected here for simplicity, provides a simple and useful quantitative tool to estimate and interpret how *T*_MI_ can be tuned toward the monitoring of oxygen rotation motions *R*_*xy*_ and *M*_*z*_ when making solid solutions^10^, applying pressure^8^, or playing with the epitaxial strain and the orientation of the substrate in thin films^29^. Our findings are relevant to other families of perovskites like A^2+^ Fe^4+^ O_3_ compounds^30^. For instance, they can explain why CaFeO_3_, which exhibits oxygen rotations, undergoes a MIT while SrFeO_3_ and BaFeO_3_, which stay cubic, remain metallic. In addition, the same physics is also inherent to manganites like LaMnO_3_, suggesting a close competition between charge and orbital orderings in this family compounds. However, the situation is slightly different in a bismutate like BaBiO_3_, in which *B*_OC_ is intrinsically unstable in the cubic phase^31^.

## Methods

### First-principles calculations

First-principles calculations were performed in the framework of DFT^32, 33^ using a Projected Augmented Wave (PAW) approach^34^ as implemented within the ABINIT package^35–38^. The calculations relied on the Generalized Gradient Approximation using the PBEsol^39^ exchange-correlation functional. We worked within a collinear spin approximation. We included a Hubbard correction *U* = 1.5 eV^40^ on the 3*d* orbitals of Ni atoms. A special care was devoted to the determination of the appropriate *U* parameter (Supplementary Notes 1– 4).

We made use of JTH atomic potentials^41^. For the wavefunctions, we used an energy cutoff of 24 Ha (38 Ha for the second grid in the PAW spheres), which guarantees a convergence better than 1 meV on the total energy. The BZ was sampled with *k*-point meshes equivalent to a 12 × 12 × 12 grid in the five-atom unit cell. During structural relaxations, thresholds of 10^−5^ Ha/bohr on the maximum force and of 10^−7^ Ha/bohr^3^ on the maximum stress have been considered.

### Structural analysis

The Goldschmidt tolerance factor^42^, *t* = *d*_R−O_/$\sqrt 2$ *d*_Ni−O_, of *R*NiO_3_ compounds were determined using Nicole Benedek’s tool^43^ relying on a bond valence model^44^ to calculate *d*_R−O_ and *d*_Ni−O_, respectively, the ideal *R*–O and Ni–O bond lengths in the cubic perovskite structure.

Symmetry-adapted mode analysis have been performed with AMPLIMODE^45, 46^. The modes are normalized to their amplitude in the *P*2_1_/*n* AFM-E′ ground state. This normalization is such that in cubic phase (volume of the ground state) *Q*_R_ = 100% corresponds to rotation angles *ϕ*_*x*_ = *ϕ*_*y*_ = 11.33° (Ni–O–Ni angle of 157.33°), *Q*_R_ = 100% corresponds to a rotation angle *ϕ*_*z*_ = 12.12° (Ni–O–Ni angle of 155.75°) and *Q*_B_ = 100% corresponds to oxygen displacements *d*_O_ = 0.0358.

### Landau model

The Landau model parameters have been fitted for YNiO_3_, GdNiO_3_, and SmNiO_3_ on first-principles data using in each case a FM cubic phase (volume of the ground state) and interpolated for the other compounds. *T*_MI_ was determined analytically (Supplementary Notes 6–9).

### Data availability

All relevant data are available from the authors.

## Electronic supplementary material

Supplementary Information
