# Supplementary material for: Structurally triggered metal-insulator transition in rare-earth nickelates
Source: Nat Commun. 2017 Nov 22;8:1677. doi: 10.1038/s41467-017-01811-x (PMC5700091; doi:10.1038/s41467-017-01811-x)
Supplement: Supplementary file 1 — Supplementary Information [file 41467_2017_1811_MOESM1_ESM.pdf]

SUPPLEMENTARY NOTE 1. VALIDATION OF THE DFT+U APPROACH

In order to assess the validity of our DFT+U approach and determine the appropriate U parameter, we have considered a wide range of possible values for U (from 0 to 8 eV) and have compared the computed structural, magnetic and electronic properties to experimental data.

The results are summarized below for $YNiO_3$ considered as a test case. In line with what was reported independently in Ref. [1], it appears that a DFT approach with a moderate U value of 1.5 eV provides for nickelates an unprecedented agreement with experimental data, combining accurate description not only of the structural but also of the magnetic and electronic properties. It therefore offers a robust and ideal framework for the study of the interplay between these properties.

SUPPLEMENTARY NOTE 2. ATOMIC STRUCTURE

In Supplementary Figure 1, we report the relative deviations respect to experimental data at low temperature [2] for the lattice parameters and atomic distortions in the E'-type AFM $P2_1/n$ phase of $YNiO_3$ in terms of the amplitude of the U parameter. The atomic distortions are those with respect to the $Pm\bar{3}m$ phase and are quantified from a symmetry-adapted mode analysis performed with AMPLIMODE [3, 4]. The labels of the modes that are allowed by symmetry in the $Pbnm$ and $P2_1/n$ phases and a brief description of the related atomic motions are reported in Supplementary Table 1.

We see in Supplementary Figure 1 that the lattice parameters are rather independent of U and well described within the whole range (error smaller than 1%). At the level of the atomic distortions, the amplitude of breathing mode $B_{\rm OC}$ (R_2^-) is only properly described in the limit of small U values. For the dominant modes like $R_{\rm xy}$ (R_5^-) or $M_{\rm z}$ (M_2^+), although the relative errors remain reasonably small for any U, the absolute amplitude evolves significantly with U and also converge to the correct values at low U.

In Supplementary Figure 2, we report comparison with experiment data [2] of the absolute amplitudes of the atomic distortions and lattice parameters in the E'-type AFM $P2_1/n$ phase of $YNiO_3$ as computed in DFT with U = 1.5 eV. It confirms that the atomic structure of $YNiO_3$ is very accurately described in DFT using PBESol and a U parameter of 1.5 eV.

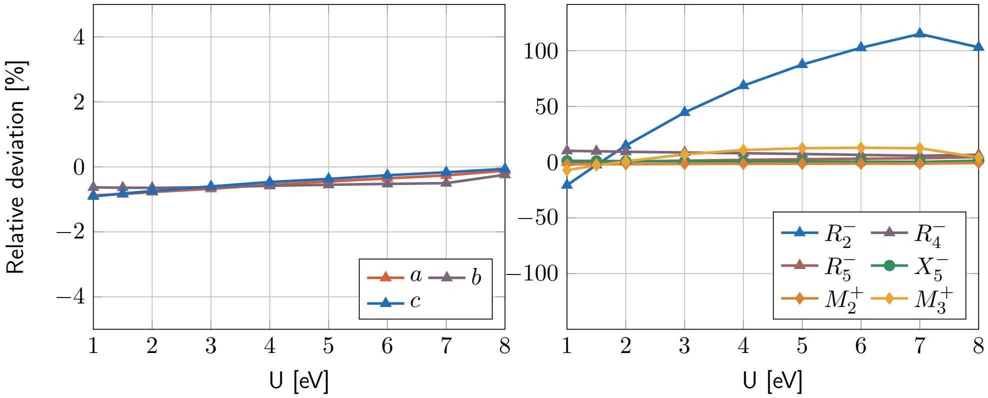

Supplementary Figure 1: **Evolution of the structural properties with U for $YNiO_3$.** Relative deviations respect to experimental data at low temperature [2] for the lattice parameters (a) and atomic distortions (b) in the E'-type AFM $P2_1/n$ phase of $YNiO_3$ in terms of the amplitude of the U parameter. Atomic distortions are quantified from a symmetry-adapted mode analysis (see Supplementary Table 1). The R_3^- and M_5^+ distortions which have negligible amplitudes ($<$ 0.05) are not shown.

Labels	Atomic motion	$Pbnm$	$P2_1/n$
R_5^-	**Anti-phase rotations of O octahedra along x and y (R_{xy})**	**x**	**x**
M_2^+	**In-phase rotations of O octahedra along z (M_z)**	**x**	**x**
X_5^-	Anti-polar (layered) motion of R cations (X_{AP})	x	x
M_3^+	Jahn-Teller distortion of O octahedra (Q_2^+)	x	x
R_4^-	Anti-polar motion (rocksalt) of R cations (R_{AP})	x	x
R_2^-	**Breathing distortion of the O octahedra (B_{OC})**		**x**
M_5^+	Anti-polar motion of O (M_{AP})		x
R_3^-	Jahn-Teller distortion of O octahedra (Q_2^-)		x

Supplementary Table I: **Structural distortions in nickelates.** Labels and description of the distortions of the $Pm\bar{3}m$ phase allowed by symmetry in the $Pbnm$ and $P2_1/n$ phases of RNiO$_3$ compounds. The main distortions are in bold.

SUPPLEMENTARY NOTE 3. MAGNETIC PROPERTIES

In order to determine the magnetic ground state of the $P2_1/n$ phase of $YNiO_3$, we performed calculations for various magnetic orders associated to supercells of up to 80 atoms.

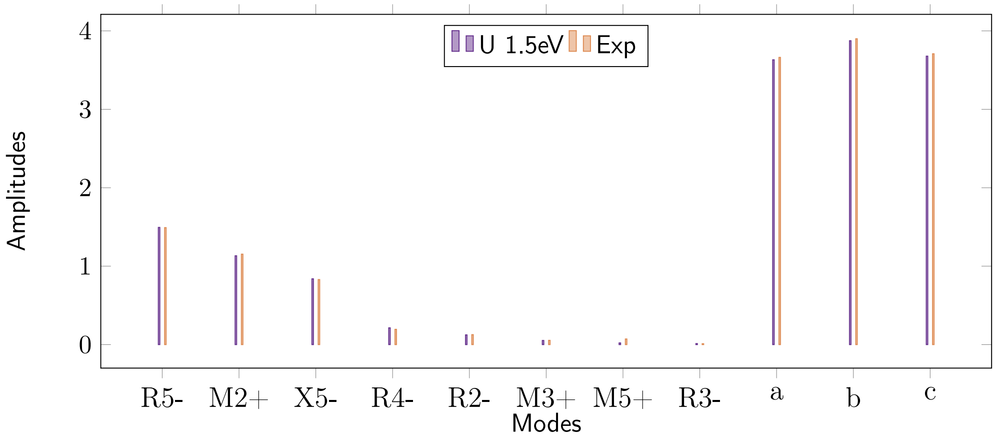

Supplementary Figure 2: **Experimental and theoretical structural properties for $YNiO_3$.** Comparison of the absolute amplitudes (Å) of the atomic distortions and lattice parameters in the E'-type AFM $P2_1/n$ phase of $YNiO_3$ as computed in DFT with U = 1.5 eV (purple) and as measured experimentally (orange). The E'-type AFM $P2_1/n$ ground state has a 80-atoms unit cell with lattice parameters $(a', b', c') = (2\sqrt{2}a, \sqrt{2}b, 4c)$.

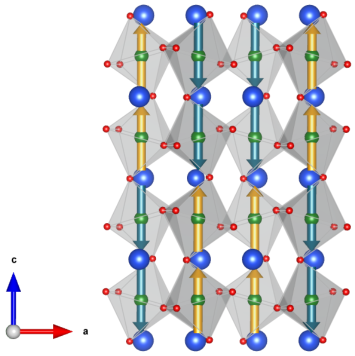

Supplementary Figure 3: **Ground State spin coniguration for $YNiO_3$.** Spin order associated to the E'-type antiferromagnetic (AFM-E') ground state.

While U values larger than 2 systematically favour a FM spin order, U = 1.5 eV properly stabilizes the E'-type spin ordering illustrated in Supplementary Figure 3 as the ground state. It corresponds to an "up-up-down-down" spin arrangement related to a Bragg vector $\mathbf{q} = (1/4, 1/4, 1/4)$ in pseudocubic notations.

In our calculations, we get a magnetic moment $\mu = 1.2\mu_B$ on the Ni atoms associated to the large oxygen octahedra and $\mu \approx 0\mu_B$ on the Ni atoms associated to the small octahedra.

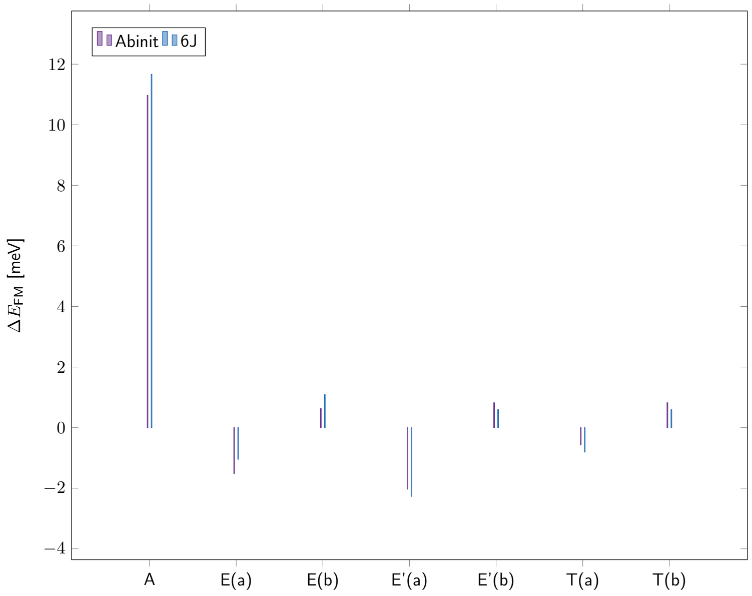

Supplementary Figure 4: **Heisenberg versus Abinit results for $YNiO_3$.** Comparison of the energy associated to various spin orders, respect to the FM order taken as reference, as obtained from our DFT calculations (purple) and simple spin model (blue). The A-type ordering corresponds to an antiferromagnetic stacking along the c direction of FM ordered ab planes. The E(a)-type order corresponds to an "up-up-down-down" zigzag chain along the a (orthorhombic) direction, and a FM order along the c direction. The E(b)-type order is characterised by the same "up-up-down-down" zigzag chain but along the b (orthorhombic) direction. The E'(a), E'(b), T(a) and T(b) types of order have the same in-plane properties too. However, the stacking along the c direction is different : E'(a) and E'(b) have two FM-order layers, AFM coupled with the next two layers while in the T(a) and T(b) configurations, the zigzag chain shifts about one spin along the chain direction at the same time one passes throught the c direction. The ground state is E'(a) as illustrated in Supplementary Figure 3 and simply called E' within the manuscript.

This is similar with what has been reported in Ref. [1, 5] and in line with the $d^8 - d^8L^2$ picture [6]. It is also compatible with experimental data as discussed in Ref. [7].

Beyond the fact that DFT calculations with U = 1.5 eV provides the right magnetic ground state, it is interesting to check if it properly accounts for the strength of the magnetic interactions. To that end, we built a simple spin model $E_{\mathrm{mag}} = (1/2) \sum J_{\mathrm{ij}} S_{\mathrm{i}} S_{\mathrm{j}}$ including J_{ij} interactions up to fourth neighbours (6 independent parameters) and fitted the parameters on our first-principles data [8]. As illustrated in Supplementary Figure 4 for $YNiO_3$ this

spin model properly reproduces the energetics of the first-principles calculations.

Monte-Carlo simulations (using large boxes up to 1728 Ni atoms) from this spin-model [8] (i) confirmed the E'-type ground state and (ii) provided a Neel temperature $T_{\mathrm{N}} = 154$ K, very similar to the mean-field estimate of 166 K and in close agreement with the experimental value of 150 K for $YNiO_3$ [9].

This demonstrates that our DFT calculations with U = 1.5 eV reproduces not only the correct E'-type magnetic ground state of nickelates but also properly describes the strength and anisotropy of their magnetic interactions.

SUPPLEMENTARY NOTE 4. ELECTRONIC PROPERTIES

Our DFT calculations with U = 1.5 eV properly accounts for the insulating character of the E'-type AFM $P2_1/n$ ground state of $YNiO_3$. For the electronic bandgap, we get a value of 0.46 eV in reasonable agreement with the experimental estimate of 0.305 eV [10].

The electronic properties are further discussed in the manuscript. As it appears clearer there, the structural and electronic properties are intimately linked together in nickelates. Hence, the fact that our simulations describe accurately the structural properties of these compounds strongly suggests that they can also be trusted to investigate their electronic properties.

SUPPLEMENTARY NOTE 5. PHONON DISPERSION CURVES

In Supplementary Figure 5, we report the full phonon dispersion curves of the $Pm\bar{3}m$ phase of $YNiO_3$, as calculated for a FM spin ordering at the volume of the $P2_1/n$ AFM-E' phase ($a_{\mathrm{pc}} = 3.728$Å). Similar curves have been obtained at the relaxed volume ($a_0 = 3.695$Å). Interpolation of these phonon dispersion curves relies on the calculation of the interatomic force constants within a $2 \times 2 \times 2$ supercell. Although this might not be totally sufficient to get a fully converged interpolation, it provides already a good estimate of the shape of the dispersion curves. It is worth to notice that the frequencies at the high-symmetry points, which are the only ones discussed below and in the manuscript, are not interpolated but calculated explicitly within our approach.

On the one hand, the phonon dispersion curves highlight strong instabilities at the R

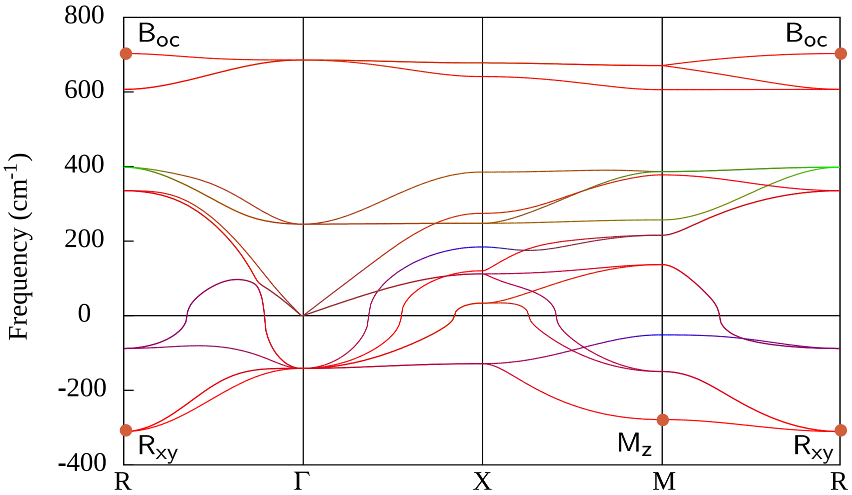

Supplementary Figure 5: **Cubic phonon dispersion curve of $YNiO_3$.** Phonon dispersion curve for the $Pm\bar{3}m$ phase of $YNiO_3$, at the $P2_1/n$ ground state volume and for a FM spin ordering (U=1.5 eV). Colors have been associated to the curves according to the involvement of each cation in the eigendisplacement of each mode (R in blue, Ni in green and O in red). Imaginary frequencies (unstable modes) appear as negative values.

and M $q-$points of the BZ, associated to the R_{xy} and M_z distortions. On the other hand, and quite amazingly, the breathing distortion, B_{OC} located at the R point, which finally produces the MIT, is associated to the hardest mode in the cubic phase. Clearly, such hard mode cannot spontaneously condense within the cubic structure.

We further notice the presence of two (triply-degenerated) unstable modes at Γ with very close frequencies. The softest one is associated to a polar-type motion involving R and O atoms; this kind of instability is expected for perovskites with small tolerance factors as $YNiO_3$. The second one is the silent butterfly motion of the O atoms.

SUPPLEMENTARY NOTE 6. LANDAU MODEL

Based on our DFT results, we have built a simple Landau-type model. In order to highlight the triggered mechanism, we restricted ourselves to the simplest possible model including only R_{xy}, M_z and B_{OC} degrees of freedom and lowest-order terms.

SUPPLEMENTARY NOTE 7. EXPRESSION

Within our Landau-type model, the free energy in terms of the amplitudes $Q_{\rm R}$, $Q_{\rm M}$ and $Q_{\rm B}$ (of $R_{\rm xy}$, $M_{\rm z}$ and $B_{\rm OC}$ respectively) reads:

$$\begin{aligned} E(Q_{\rm M}, Q_{\rm R}, Q_{\rm B}) =& \alpha_{\rm R} Q_{\rm R}^2 + \beta_{\rm R} Q_{\rm R}^4 + \alpha_{\rm M} Q_{\rm M}^2 + \beta_{\rm M} Q_{\rm M}^4 + \alpha_{\rm B} Q_{\rm B}^2 + \beta_{\rm B} Q_{\rm B}^4 \\ &+ \lambda_{\rm MR} Q_{\rm M}^2 Q_{\rm R}^2 + \lambda_{\rm MB} Q_{\rm M}^2 Q_{\rm B}^2 + \lambda_{\rm RB} Q_{\rm R}^2 Q_{\rm B}^2 \end{aligned} \quad (1)$$

The parameters $\alpha_{\rm R}$ and $\alpha_{\rm M}$ are assumed to be temperature dependent as

$$\alpha_{\rm R} = \gamma_{\rm R}(T_{\rm 0R} - T) \quad \text{and} \quad \alpha_{\rm M} = \gamma_{\rm M}(T_{\rm 0M} - T) \quad (2)$$

while all the other parameters are supposed to be constant.

Other modes allowed by symmetry (see Supplementary Table 1) in the $Pbnm$ and $P2_1/n$ phases have not be explicitly included within the model. Some of them, like X_5^- (and to a lesser extent R_4^- and M_3^+), take however a significant amplitude and are crucial to stabilize the $Pbnm$ phase. They are implicitly included through a renormalization of the $\lambda_{\rm MR}$ parameter as it will appear more clearly in the next section.

The strain degrees of freedom have not been explicitly included within the model to highlight the key role of phonon-phonon couplings, which appear sufficient to reproduce experimental data on bulk compounds. However, this model could be naturally extended to strain degrees of freedom and their couplings with lattice modes. This might be useful to quantify for instance the role of epitaxial strain in thin films but is beyond the scope of this work.

The expansion has been limited to 4th order for all three order parameters including $Q_{\rm B}$. This is justified by the fact that, from the fit of the parameters, the triggered transition appears to be second order . We notice however that explicit treatment of the strain (neglected here) could affect the order of the phase transition as further discussed below.

SUPPLEMENTARY NOTE 8. FIT FROM DFT

Parameters of our Landau-type model have been fitted on first-principles results. At first, we focused on $YNiO_3$.

We considered in our calculations a fixed cubic $Pm\bar{3}m$ cell at a volume similar to that of the $P2_1/n$ AFM-E' ground-state ($a_{\rm pc} = 3.728$Å), which corresponds to imposing a negative

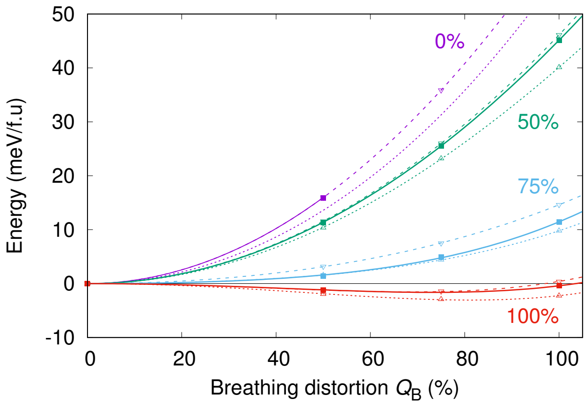

Supplementary Figure 6: **Breathing mode in $YNiO_3$ as a function of rotation modes.** Evolution of the energy of $YNiO_3$ in terms of the amplitude of the breathing distortion for fixed amplitude of oxygen rotations ($Q_R = Q_M$, from 0% to 100%) in a FM configuration and either a fixed cubic cell (dotted line and triangles: $a_{pc} = 3.728$ Å; dashed line and back triangles: $a_0 = 3.695$ Å) or when relaxing fully the lattice parameters (full line and squares).

strain of 0.9%. At this volume, relaxing within the $P2_1/n$ symmetry while keeping the unit cell fixed yields amplitudes of distortion comparable to the ground-state. We notice that, as illustrated in Supplementary Figure 6, similar calculations performed at the relaxed lattice constant ($a_0 = 3.695$Å) yield very similar results. Even calculations performed while relaxing the lattice parameters at fixed mode amplitudes (in reduced coordinates) do not provide any significant change.

The calculations have been performed with a FM spin order which does not break any symmetry. We checked explicitly that the key physical features and conclusions (cooperative bi-quadratic coupling between rotations and breathing and triggered mechanism) remain similar for different AFM spin orders. The results remain even very similar in a non-magnetic (NM) calculation (with or without U correction) although, in that case, the amplitude of rotations required to destabilise B_{OC} is slightly larger ($\approx 160\%$); this last result illustrates that electronic Hund's rule energy, although playing a role, is not driving alone the appearance of B_{OC} as sometimes suggested [11].

The parameters of the Landau model at 0 K have then been extracted from DFT data as follows.

- $\alpha^0_{\rm R}(=\gamma_{\rm R}T_{\rm 0R})$, $\alpha^0_{\rm M}(=\gamma_{\rm M}T_{\rm 0M})$, $\beta_{\rm R}$ and $\beta_{\rm M}$ were fitted on the individual double wells associated to $R_{\rm xy}$ and $M_{\rm z}$ (Figure 1).

- $\lambda_{\rm RM}$ was fitted to reproduce the energy of a relaxed *Pbnm*-like phase (full atomic relaxation while keeping the cubic cell fixed). From this, we renormalize the natural competition between $R_{\rm xy}$ and $M_{\rm z}$ by including implicitly the stabilising effect of X_5^-, R_4^- and M_3^+ modes. We notice that in all compounds, $R_{\rm xy}$ and $M_{\rm z}$ compete with each other and should yield $\lambda_{\rm RM} > 0$. However, because of the renormalization due to the implicit presence of the other modes, $\lambda_{\rm RM}$ becomes negative for large cations (i.e. X_5^- helps stabilizing the *Pbnm* phase consistently with the discussion in Ref. [12].

- $\alpha_{\rm B}$ was fitted on the single well associated to $B_{\rm OC}$ (Figure 1).

- $\lambda_{\rm BR}$ and $\lambda_{\rm BM}$ were fitted from the change of curvature of the well of $B_{\rm OC}$ when freezing 100% of $Q_{\rm R}$ and $Q_{\rm M}$ respectively (Figure 2).

- $\beta_{\rm B}$ was fitted to reproduce the right amplitude of $B_{\rm OC}$ in the ground state of the model and it was checked that the result still properly describes the single well associated to $B_{\rm OC}$.

Within the model, the amplitude for the atomic distortion are renormalised to the one obtained from DFT calculation for the $YNiO_3$ ground state. This means 1 for rotation, tilts and breathing mode correspond to the amplitude of these modes in a cubic box with lattice parameters coresponding to 3.728 Å.

We applied the same procedure to $GdNiO_3$ and $SmNiO_3$. All the computed parameters are summarized in Table II.

As illustrated in Supplementary Figure 7, all the parameters have an almost linear dependence in terms of the tolerance factor t. So, in our model, we assumed such a linear dependence to determine the value of the parameters at arbitrary t.

Finally, knowing $\alpha_{\rm R,M}$ at 0 K (from the DFT calculations), their temperature dependence was estimated as follows. Focusing first on $YNiO_3$ we adjusted $T_{\rm 0R} = T_{\rm 0M}$ so that within our model, $B_{\rm OC}$ appears at the experimental value of 585K [13] and we deduced $\gamma_{\rm M,R} = \alpha^0_{\rm R,M}/T_{\rm 0R,M}$: $\gamma_{\rm M} = 0.148 meV/f.u.$ and $\gamma_{\rm R} = 0.331 meV/f.u.$. Then, assuming $\gamma_{\rm M,R}$ constant within the whole family the evolution of $T_{\rm 0R}$ and $T_{\rm 0M}$ with the tolerance factor were obtained as: $T_{\rm 0R,M} = \alpha^0_{\rm R,M}/\gamma_{\rm M,R}$.

From this, we get the following final Landau-type expression for the free energy, allowing

parameter	Y	Gd	Sm
t	0.920	0.938	0.947
α_{B}	58.1	52.6	50.6
β_{B}	10.0	16.0	31.0
α^0_{M}	-578.3	-385.5	-277.3
$T_{0\mathrm{M}}$	3918	2571	1897
β_{M}	213.9	209.4	201.0
α^0_{R}	-1288.1	-1081.2	-920.9
$T_{0\mathrm{R}}$	3918	3195	2833
β_{R}	648.8	754.9	750.4
λ_{MR}	31.8	-57.7	-99.4
λ_{MB}	-26.0	-29.0	-29.4
λ_{RB}	-35.8	-42.2	-42.4

Supplementary Table II: **Fitted Landau parameters as a function of the tolerance factor.** Landau model parameters (meV/f.u. or K) as fitted on first-principles data, using the mode normalisation described in the Methods section.

us to determine the values of Q_{R},Q_{M} and Q_{B} for any value of the temperature T and tolerance factor t :

$$
\begin{aligned}
&E(Q_{\mathrm{M}}, Q_{\mathrm{R}}, Q_{\mathrm{B}}) = \\
&(-281.9 \times t + 317.4)Q_{\mathrm{B}}^2 + (714.3 \times t - 648.9)Q_{\mathrm{B}}^4 \\
&- 0.15 \times (-74891.2 \times t + 72818.6 - T) \times Q_{\mathrm{M}}^2 + (-446.3 \times t + 625.3) \times_{\mathrm{M}}^4 \\
&- 0.33 \times (-40212.2 \times t + 40913.9 - T) \times Q_{\mathrm{R}}^2 + (4067.9 \times t - 3085.5) \times Q_{\mathrm{R}}^4 \\
&+ (-4875.7 \times t + 4517)Q_{\mathrm{M}}^2 Q_{\mathrm{R}}^2 + (-131.6 \times t + 94.9) \times Q_{\mathrm{M}}^2 Q_{\mathrm{B}}^2 + (-258.6 \times t + 201.6) \times Q_{\mathrm{R}}^2 Q_{\mathrm{B}}^2
\end{aligned}
\tag{3}
$$

SUPPLEMENTARY NOTE 9. ANALYTICAL SOLUTION

From this model, T_{MI} can be determined analytically.

We start from the expression:

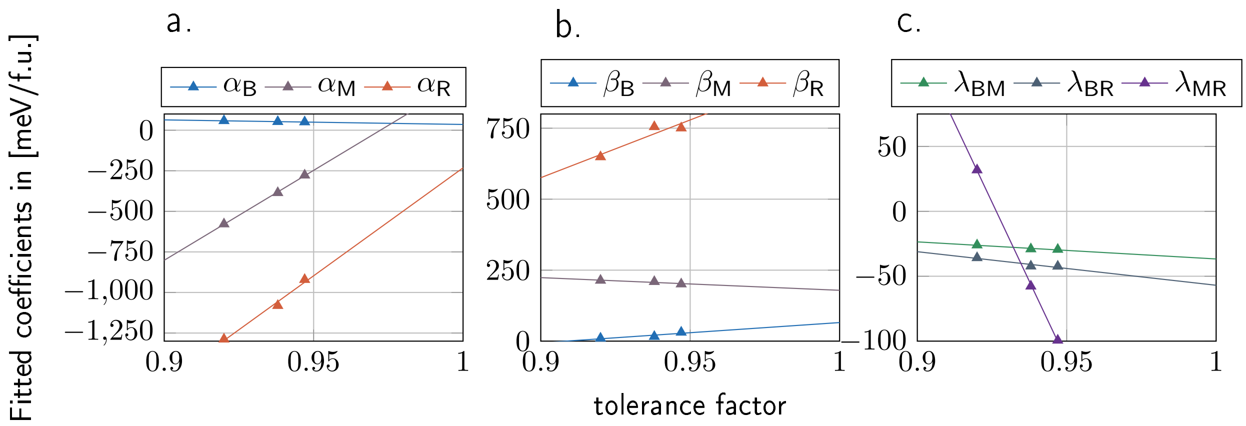

Supplementary Figure 7: **Landau parameters as a function of the tolerance factor.** Evolution of the model parameters (meV/f.u.) with the tolerance factor.

$$\begin{aligned} E(Q_\mathrm{M}, Q_\mathrm{R}, Q_\mathrm{B}) = &\quad \alpha_\mathrm{B} Q_\mathrm{B}^2 + \beta_\mathrm{B} Q_\mathrm{B}^4 \\ &+ \gamma_\mathrm{M} \times (T_{0\mathrm{M}} - T) \times Q_\mathrm{M}^2 + \beta_\mathrm{M} Q_\mathrm{M}^4 \\ &+ \gamma_\mathrm{R} \times (T_{0\mathrm{R}} - T) \times Q_\mathrm{R}^2 + \beta_\mathrm{R} Q_\mathrm{R}^4 \\ &+ \lambda_\mathrm{MR} Q_\mathrm{M}^2 Q_\mathrm{R}^2 + \lambda_\mathrm{MB} Q_\mathrm{M}^2 Q_\mathrm{B}^2 + \lambda_\mathrm{RB} Q_\mathrm{R}^2 Q_\mathrm{B}^2 \end{aligned} \quad (4)$$

At the energy minimum, we should have :

$$\frac{\partial E}{\partial Q_\mathrm{M}} = 0 \text{ and } \frac{\partial E}{\partial Q_\mathrm{R}} = 0. \quad (5)$$

The solutions for that, other than $Q_\mathrm{M} = 0$ and $Q_\mathrm{R} = 0$, are :

$$\begin{aligned} Q_\mathrm{M}^2 &= \frac{-2T_{0\mathrm{M}}\gamma_\mathrm{M}\beta_\mathrm{R} + T_{0\mathrm{R}}\gamma_\mathrm{R}\lambda_\mathrm{MR} + T(2\gamma_\mathrm{M}\beta_\mathrm{R} - \gamma_\mathrm{R}\lambda_\mathrm{MR})}{4\beta_\mathrm{M}\beta_\mathrm{R} - \lambda_\mathrm{MR}^2} \\ &+ \frac{(-2\beta_\mathrm{R}\lambda_\mathrm{MB} + \lambda_\mathrm{MR}\lambda_\mathrm{RB})}{4\beta_\mathrm{M}\beta_\mathrm{R} - \lambda_\mathrm{MR}^2} Q_\mathrm{B}^2 \\ Q_\mathrm{R}^2 &= \frac{-2T_{0\mathrm{R}}\gamma_\mathrm{R}\beta_\mathrm{M} + T_{0\mathrm{M}}\gamma_\mathrm{M}\lambda_\mathrm{MR} + T(2\gamma_\mathrm{R}\beta_\mathrm{M} - \gamma_\mathrm{M}\lambda_\mathrm{MR})}{4\beta_\mathrm{M}\beta_\mathrm{R} - \lambda_\mathrm{MR}^2} \\ &+ \frac{(-2\beta_\mathrm{M}\lambda_\mathrm{RB} + \lambda_\mathrm{MR}\lambda_\mathrm{MB})}{4\beta_\mathrm{M}\beta_\mathrm{R} - \lambda_\mathrm{MR}^2} Q_\mathrm{B}^2 \end{aligned} \quad (6)$$

Introducing this in Supplementary Eq. (4) we get :

$$E(Q_\mathrm{B}) = \alpha'_\mathrm{B} Q_\mathrm{B}^2 + \beta'_\mathrm{B} Q_\mathrm{B}^4 \quad (7)$$

where

$$\alpha'_{\mathrm{B}} = \alpha_{\mathrm{B}} + \tfrac{(\gamma_{\mathrm{M}}\lambda_{\mathrm{MR}}\lambda_{\mathrm{RB}} - 2\gamma_{\mathrm{M}}\beta_{\mathrm{R}}\lambda_{\mathrm{MB}})}{4\beta_{\mathrm{M}}\beta_{\mathrm{R}} - \lambda_{\mathrm{MR}}^2}(T_{0\mathrm{M}} - T) + \tfrac{(\gamma_{\mathrm{R}}\lambda_{\mathrm{MB}}\lambda_{\mathrm{MR}} - 2\gamma_{\mathrm{R}}\beta_{\mathrm{M}}\lambda_{\mathrm{RB}})}{4\beta_{\mathrm{M}}\beta_{\mathrm{R}} - \lambda_{\mathrm{MR}}^2}(T_{0\mathrm{R}} - T) \tag{8}$$

$$\beta'_{\mathrm{B}} = \beta_{\mathrm{B}} + \tfrac{\lambda_{\mathrm{MB}}\lambda_{\mathrm{MR}}\lambda_{\mathrm{RB}} - \beta_{\mathrm{M}}\lambda_{\mathrm{RB}}^2 - \beta_{\mathrm{R}}\lambda_{\mathrm{MB}}^2}{4\beta_{\mathrm{M}}\beta_{\mathrm{R}} - \lambda_{\mathrm{MR}}^2} \tag{9}$$

$$\tag{10}$$

The MIT is linked to the appearance of the B_{OC}. This will appear at a temperature T_{MI} at which $\alpha'_{\mathrm{B}} = 0$. This critical temperature is given by :

$$T_{\mathrm{MI}} = \tfrac{\alpha_{\mathrm{B}}(-4\beta_{\mathrm{M}}\beta_{\mathrm{R}} + \lambda_{\mathrm{MR}}^2)}{2\gamma_{\mathrm{M}}\beta_{\mathrm{R}}\lambda_{\mathrm{MB}} - \gamma_{\mathrm{R}}\lambda_{\mathrm{MB}}\lambda_{\mathrm{MR}} + 2\gamma_{\mathrm{R}}\beta_{\mathrm{M}}\lambda_{\mathrm{RB}} - \gamma_{\mathrm{M}}\lambda_{\mathrm{MR}}\lambda_{\mathrm{RB}}} + \tfrac{T_{0\mathrm{R}}\gamma_{\mathrm{R}}(-\lambda_{\mathrm{MB}}\lambda_{\mathrm{MR}} + 2\beta_{\mathrm{M}}\lambda_{\mathrm{RB}})}{2\gamma_{\mathrm{M}}\beta_{\mathrm{R}}\lambda_{\mathrm{MB}} - \gamma_{\mathrm{R}}\lambda_{\mathrm{MB}}\lambda_{\mathrm{MR}} + 2\gamma_{\mathrm{R}}\beta_{\mathrm{M}}\lambda_{\mathrm{RB}} - \gamma_{\mathrm{M}}\lambda_{\mathrm{MR}}\lambda_{\mathrm{RB}}} + \tfrac{T_{0\mathrm{M}}\gamma_{\mathrm{M}}(2\beta_{\mathrm{R}}\lambda_{\mathrm{MB}} - \lambda_{\mathrm{MR}}\lambda_{\mathrm{RB}}))}{2\gamma_{\mathrm{M}}\beta_{\mathrm{R}}\lambda_{\mathrm{MB}} - \gamma_{\mathrm{R}}\lambda_{\mathrm{MB}}\lambda_{\mathrm{MR}} + 2\gamma_{\mathrm{R}}\beta_{\mathrm{M}}\lambda_{\mathrm{RB}} - \gamma_{\mathrm{M}}\lambda_{\mathrm{MR}}\lambda_{\mathrm{RB}}} \tag{11}$$

Furthermore, supposing a linear dependence for all the coefficients with respect to the tolerance factor, we get a generic expression :

$$T_{\mathrm{MI}} = \frac{a + t \times (b + (c + d \times t)t)}{e + t \times (f + g \times t)} \tag{12}$$

where t is the tolerance factor and a, b, c, d, e, f and g are a combination of model parameters.

Using the coefficients determined from DFT calculations in the previous Section, we can predict the evolution of T_{MI} as a function of the tolerance factor as illusrated in Figure 2b, blue line.

Independently, we can also fit the experimental data point using Supplementary Eq. 13. Making such a fit, while excluding Nd and Pr compounds, we get the dashed blue line in Figure 2b.

SUPPLEMENTARY NOTE 10. ORDER OF THE TRANSITION

Experimentally, there is still some debate about the order of the MIT. For large cations ($T_{\mathrm{MI}} = T_{\mathrm{N}}$), the MIT is rather abrupt and hysteretic and unanimously considered as being first order [13]. The magnetic transition that takes place at the same temperature is also first order [14]. For small cations ($T_{\mathrm{MI}} > T_{\mathrm{N}}$), the MIT is less hysteretic and sometimes considered as evolving to second-order. Some studies seem however to show that it stays first-order

[15, 16], while the less hysteretic behavior could be related to the fact that kinetics are better at higher temperatures [13]. For these compounds the magnetic transition is second-order.

As previously mentioned, the MIT is predicted to be second-order within our very simple model. As highlighted in Supplementary Table 2, computed β_{B} is positive for all compounds. From Supplementary Eq. 10, the oxygen rotations renormalize the fourth-order term coefficient and a negative value of β'_{B} would give rise to a first-order transition (it would then further require including 6th order terms in Q_{B}). Although this renormalization is negative, β'_{B} stays nevertheless positive in all cases ($\beta'_{\mathrm{B}} = 9$, 14 and 29 respectively for $YNiO_3$, $GdNiO_3$ and $SmNiO_3$ respectively) corresponding therefore to a second-order transition.

Yet, we have to stress that our approach does not allow us to address the order of the transition conclusively. First, our model is built at fixed cubic cell and does not include strain relaxation. Explicit treatment of the latter will further renormalize the 4th-order term and might potentially make it negative, so eventually changing the order of the transition. Second, at a more fundamental level, even if our DFT+U results suggest that Q_{B} undergoes a second-order transition, that does not rule out the possibility that thermal effects effectively render a first-order transformation driven by temperature. The ferroelectric phase transitions of $BaTiO_3$, a well studied case, are a concrete example of this [17], and also illustrate the critical role of strains to enhance the discontinuous character of the transformation [18]. Hence, discussing the character of the transition from first-principles would require explicitly statistical simulations that fall beyond of the scope of this work. The Landau model introduced here was kept simple on purpose in order to highlight the key role of the triggered mechanism and it show that, based on our first-principles results and a minimal experimental input, the main features of the phase diagram can be readily reproduced. Further, the main trends (regarding ionic size, amplitude of the different rotations, ...) are properly captured by this simple model, which moreover provides us with insights about how to tune the behaviour of these materials. In this sense, we consider that the proposed Landau model is valid and useful although, admittedly, it is not suitable for a definite discussion of the order of the transition.

For large cations (Nd and Pr), our model predicts that the MIT can no more be fully triggered by the oxygen rotations (which are reduced); for those compounds, it is complementarily promoted by the appearance of the AFM-E' magnetic order. In such case, the MIT takes place at $T_{\mathrm{MI}} = T_{\mathrm{N}}$; it is expected to be more abrupt and to be first order. Such

coupling between the structural and magnetic transition for large cations is in line with the conclusions of Vobornik *et al.* in Ref. [14]: they suggest indeed that, contrary to other cases, there is a possible interplay between electronic and magnetic degrees of freedom when $T_{\mathrm{MI}} = T_{\mathrm{N}}$ and that any further model of the TMI should address that fact. Our manuscript explicitly addresses that point and we believe that it convincingly answers their questioning.

SUPPLEMENTARY NOTE 11. ELECTRONIC BAND STRUCTURES

In Supplementary Figure 8, we report the electronic dispersion curves of $YNiO_3$ with a FM spin order, along a more exhaustive path of the Brillouin zone of the $Pbnm$ or $P2_1/n$ 20-atom cell. The majority spins are in colors while the minority spins are in light grey. The latter have been omitted for clarity in the main manuscript. We notice that the cubic phase is essentially non magnetic (up and down spin bands nearly degenerate) and magnetism starts to develop with the rotations.

In Supplementary Figure 9, we report similarly the electronic dispersion curves of $YNiO_3$ but with an AFM-A spin order. This figure is very similar to the previous one, demonstrating that our results are not dependent of the specific choice of spin order.

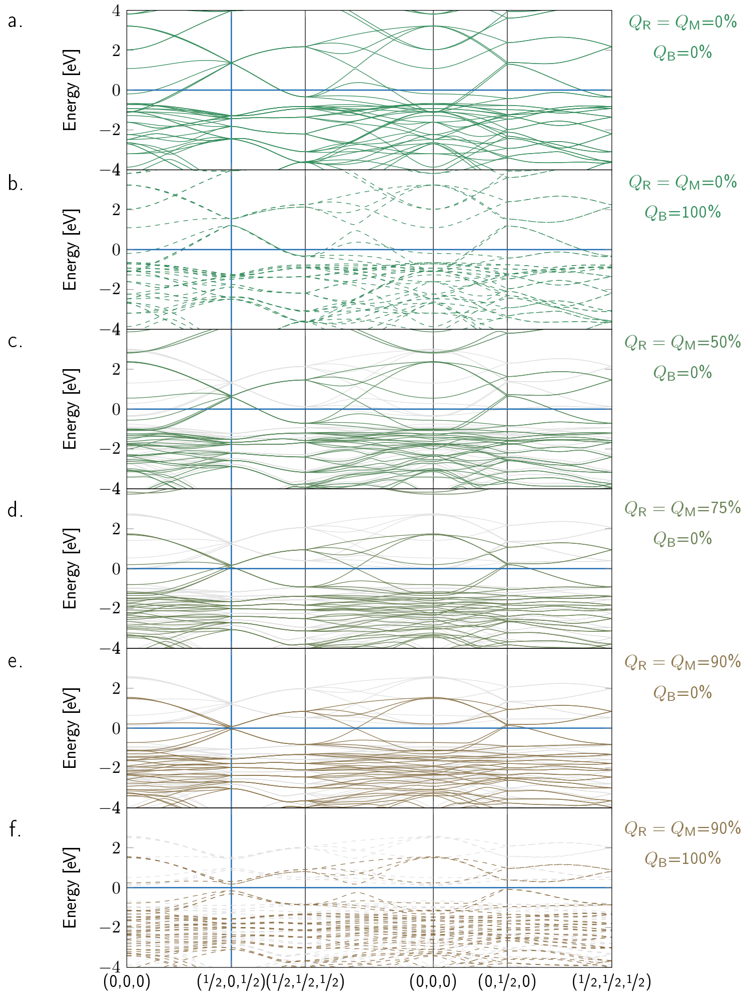

Supplementary Figure 8: **$YNiO_3$ FM electronic band structures.** $YNiO_3$, FM spin order. Evolution of the electronic dispersion curves around the Fermi energy E_f when freezing into the $Pm\bar{3}m$ phase increasing amplitudes of oxygen rotations ($Q_R = Q_M =$ (**a**) 0%, (**c**) 50%, (**d**) 75% and (**e**) 90%, lines) and eventually adding the breathing distortion ($Q_B = 100\%$ with $Q_R = Q_M =$ (**b**) 0% and (**f**) 90%, dashed lines). The graph connects high-symmetry points in the Brillouin zone of the $Pbnm$ or $P2_1/n$ 20-atom cell. Majority spins are in colors and minority spins in light grey.

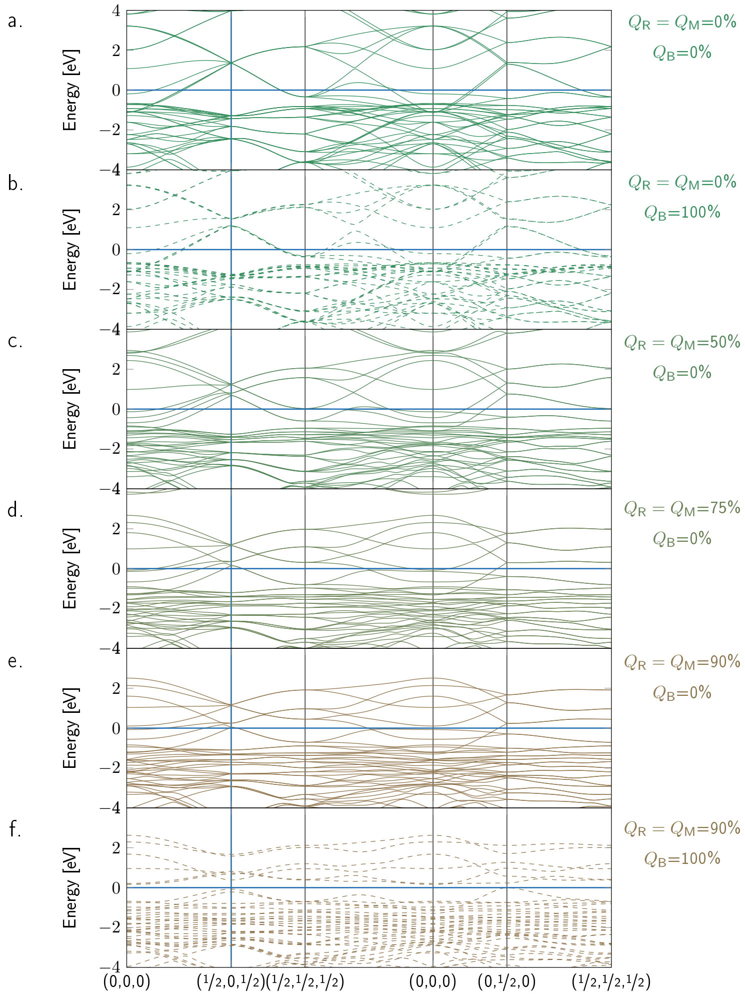

Supplementary Figure 9: **$YNiO_3$ AFM-A electronic band structures.** $YNiO_3$, AFM-A spin order. Evolution of the electronic dispersion curves around the Fermi energy E_f when freezing into the $Pm\bar{3}m$ phase increasing amplitudes of oxygen rotations ($Q_R = Q_M =$ (**a**) 0%, (**c**) 50%, (**d**) 75% and (**e**) 90%, lines) and eventually adding the breathing distortion ($Q_B = 100\%$ with $Q_R = Q_M =$ (**b**) 0% and (**f**) 90%, dashed lines). The graph connects high-symmetry points in the Brillouin zone of the $Pbnm$ or $P2_1/n$ 20-atom cell.

SUPPLEMENTARY REFERENCES

[1] Varignon, J., Grisolia, M. N., Iniguez, J., Barthélémy, A. & Bibes, M. Complete phase diagram of rare-earth nickelates from first principles. *npj Quantum Materials* **2**, 21 (2017).

[2] Alonso, J. A. *et al.* High-temperature structural evolution of R NiO 3 (R = H o , Y , E r , Lu) perovskites: Charge disproportionation and electronic localization. *Physical Review B* **64** (2001).

[3] Orobengoa, D., Capillas, C., Aroyo, M. I. & Perez-Mato, J. M. *AMPLIMODES*: symmetry-mode analysis on the Bilbao Crystallographic Server. *Journal of Applied Crystallography* **42**, 820–833 (2009).

[4] Perez-Mato, J. M., Orobengoa, D. & Aroyo, M. I. Mode crystallography of distorted structures. *Acta Crystallographica Section A* **66**, 558–590 (2010).

[5] Prosandeev, S., Bellaiche, L. & Íñiguez, J. Ab initio study of the factors affecting the ground state of rare-earth nickelates. *Physical Review B* **85**, 214431 (2012).

[6] Johnston, S., Mukherjee, A., Elfimov, I., Berciu, M. & Sawatzky, G. A. Charge Disproportionation without Charge Transfer in the Rare-Earth-Element Nickelates as a Possible Mechanism for the Metal-Insulator Transition. *Physical Review Letters* **112**, 106404 (2014).

[7] Muñoz, A., Alonso, J. A., Martínez-Lope, M. J. & Fernández-Díaz, M. T. On the magnetic structure of DyNiO3. *Journal of Solid State Chemistry* **182**, 1982–1989 (2009).

[8] Mercy, A., J., B. & Ghosez, P. Magnetic properties of $YNiO_3$. *unpublished* (2017).

[9] Alonso, J. A. *et al.* Charge Disproportionation in $RNiO_3$ Perovskites: Simultaneous Metal-Insulator and Structural Transition in $YNiO3$. *Physical Review Letters* **82**, 3871–3874 (1999).

[10] Arima, T., Tokura, Y. & Torrance, J. B. Variation of optical gaps in perovskite-type 3 d transition-metal oxides. *Physical Review B* **48**, 17006–17009 (1993).

[11] Mazin, I. I. *et al.* Charge Ordering as Alternative to Jahn-Teller Distortion. *Physical Review Letters* **98**, 176406 (2007).

[12] Benedek, N. A. & Fennie, C. J. Why are there so few perovskite ferroelectrics? *The Journal of Physical Chemistry C* **117**, 13339–13349 (2013).

[13] Catalan, G. Progress in perovskite nickelate research. *Phase Transitions* **81**, 729–749 (2008).

[14] Vobornik, I. *et al.* Electronic-structure evolution through the metal-insulator transition in *$RNiO_3$*. *Phys. Rev. B* **60**, R8426–R8429 (1999).

[15] Perez-Cacho, J., Blasco, J., Garcia, J., Castro, M. & Stankiewicz, J. Study of the phase transitions in $SmNiO_3$. *J. Phys.: Condens. Matter* **11**, 405 (1999).

[16] Nikulina, I., Novojilova, M., Kaulb, A., Maiorovab, A. & S.N., M. Synthesis and transport properties study of $Nd_{1-x}Sm_xNiO_{3-\delta}$ solid solutions. *Materials Research Bulletin* **39**, 803 (2004).

[17] Iniguez, J., Ivantchev, S., Perez-Mato, J. M. & Garcia, A. Devonshire-landau free energy of $BaTiO_3$ from first principles. *Phys. Rev. B* **63**, 144103 (2001).

[18] Zhong, W., Vanderbilt, D. & Rabe, K. M. First-principles theory of ferroelectric phase transitions for perovskites: The case of $BaTiO_3$. *Phys. Rev. B* **52**, 6301–6312 (1995).
